# A Digital Health Application Allowing a Personalized Low-Glycemic Nutrition for the Prophylaxis of Migraine: Proof-of-Concept Data from a Retrospective Cohort Study

**DOI:** 10.3390/jcm11041117

**Published:** 2022-02-20

**Authors:** Torsten Schröder, Gianna Kühn, Anna Kordowski, Soodeh Razeghi Jahromi, Astrid Gendolla, Stefan Evers, Charly Gaul, Diamant Thaçi, Inke Regina König, Christian Sina

**Affiliations:** 1 Institute of Nutritional Medicine, University Hospital of Schleswig-Holstein, Campus Lübeck & University of Lübeck, Ratzeburger Allee 160, 23538 Lübeck, Germany; torsten.schroeder@perfood.de (T.S.); anna.kordowski@uksh.de (A.K.); 2 Perfood GmbH, Research & Development, Am Spargelhof 2, 23554 Lübeck, Germany; gianna.kuehn@perfood.de; 3 Department of Clinical Nutrition and Dietetics, Faculty of Nutrition and Food Technology, Shahid Beheshti University of Medical Sciences, Farahzadi Blvd, Hafezi Str. 3, Tehran 1981619537, Iran; soodehrazeghi@gmail.com; 4 Medical Practice for Neurology and Pain Therapy Essen, Am Alfredusbad 2, 45133 Essen, Germany; a.gendolla@praxis-gendolla.de; 5 Faculty of Medicine, University of Münster, Domagkstr. 3, 48149 Münster, Germany; everss@uni-muenster.de; 6 Department of Neurology, Krankenhaus Lindenbrunn, 31863 Coppenbrügge, Germany; 7 Headache Center Frankfurt, Dalbergstr. 2a, 65929 Frankfurt am Main, Germany; c.gaul@kopfschmerz-frankfurt.de; 8 Institute and Comprehensive Center for Inflammation Medicine, University of Lübeck, Ratzeburger Allee 160, 23538 Lübeck, Germany; diamant.thaci@uksh.de; 9 Institute of Medical Biometry and Statistics, University Hospital of Schleswig-Holstein, Campus Lübeck & University of Lübeck, Ratzeburger Allee 160, 23538 Lübeck, Germany; inke.koenig@uni-luebeck.de; 10 Medical Department 1, Section of Nutritional Medicine University Hospital of Schleswig-Holstein, Campus Lübeck & University of Lübeck, Ratzeburger Allee 160, 23538 Lübeck, Germany

**Keywords:** migraine prophylaxis, personalized nutrition, continuous glucose measurement, digital health application, low-glycemic diet, low-glycemic index, headache, diet

## Abstract

Background: Migraine is a headache disorder with the highest socioeconomic burden. The aim of this study was to deliver the first proof-of-concept data of the potential role of an individual low-glycemic diet provided by a novel digital health application in the prophylaxis of migraine. Methods: We analyzed data from a retrospective survey of individuals who participated in a digital nutrition program that provides dietary recommendations based on the individual analysis of continuous glucose measurement from an up to 14-day test phase. A total of 84 individuals completed the retrospective digital survey. The endpoints were changes in the number of migraine days, average duration of attacks, average pain severity, frequency of intake of pain medication, absenteeism, and presenteeism before and after program participation. Results: The intraindividual comparisons of the endpoints before and after program participation revealed decreases in migraine frequency and other patient-relevant migraine parameters. Moreover, patients with a baseline migraine frequency of two and more migraine days per month and adherence to the dietary recommendations (*n* = 40) showed a mean reduction in migraine days by 33% with a 50%-responder rate of 38%. Conclusions: The data provides emerging evidence that an individualized low-glycemic diet based on continuous glucose measurement could be a promising approach for a diet-based, non-pharmacological migraine prophylaxis. However, future research is required to confirm the implied effectiveness.

## 1. Introduction

Migraine is a highly disabling headache disorder with an estimated global prevalence of around 15% of the world’s population [1,2,3,4]. Migraine is associated with a high individual burden and is considered a major socioeconomic problem with approximately $36 billion in direct and indirect medical costs in the United States alone [2,5,6].

For migraine with frequent attacks, severe accompanying symptoms, persistent aura, or insufficient effect of acute medication, migraine prophylaxis should be offered to the patient, according to the guidelines [7,8]. The pharmacological prophylaxis of migraine attacks is usually carried out with drugs originally intended for other indications, such as beta blockers, calcium channel blockers, anticonvulsants, or antidepressants, as these drugs have an “incidental” effect that reduces migraine frequency [7,9,10]. However, most of these drugs may lead to numerous adverse events such as dizziness, diarrhea, fatigue, weight gain, or erectile dysfunction, which is believed to be the main reason that drug adherence is very low [11,12]. A relatively new—and first migraine specific—pharmacological strategy is the blockade of the neuropeptide calcitonin gene-related peptide (CGRP) or its receptor by antibodies [13]. However, due to high costs, this approach is currently not routinely offered to all patients [14,15]. An accompanying non-pharmacological therapy and prophylaxis are frequently recommended and also mentioned in some clinical guidelines [7]. For instance, these non-pharmacological approaches include, above all, stress-reducing measures such as muscle relaxation or biofeedback, acupuncture, neurostimulation, and nutritional supplements [16]. However, non-pharmacological therapy is not yet part of standardized migraine therapy and is not accessible in all parts of the world. In addition, specific dietary interventions are not part of the current standard of care, although more than two-thirds of all migraine patients report their diet as a trigger on migraine activity, such as prolonged periods of fasting, alcohol, or distinct food [17]. In particular, certain foods such as cheese, chocolate, or citrus fruits are frequently reported to induce migraine attacks [18,19]. However, there is currently no clear understanding of a possible relation and usually, provocation studies fail [20]. Consequently, many authors recommend that clinicians should refrain from supporting an appreciation that avoidance of specific food items had a role in therapy as long as studies have not found sufficient supporting evidence [10].

Potentially greater influence than food triggers may be body weight and obesity-related metabolism. In a recent meta-analysis including a total of 10 randomized studies, a positive effect of weight reduction could be demonstrated [21]. However, the underlying mechanisms remain uncertain since the reduction in migraine activity was independent of the amount of body weight loss.

A potential pathological link between nutrition and migraine may be a disturbed glucose-insulin metabolism. Glucose has an essential role in the energy supply to the central nervous system and was hypothesized to be a potential pathophysiological correlate for migraine as early as 1935 [22]. Recent data suggest that energy and particularly glucose-dependent mechanisms play a role in migraine pathology including cortical spreading depolarization and TRPA1-induced CGRP release [23]. Supporting evidence for an important role of glucose-insulin metabolism comes from a recent dietary intervention trial demonstrating that a very low carbohydrate diet, which is known to reduce the overall glycemic load, significantly improved migraine symptoms in patients with drug-refractory chronic migraine [24]. Moreover, Evcili et al. studied the effect of a low-glycemic index (GI) diet compared with migraine prophylaxis with either propranolol, flunarizine, or amitriptyline in a cohort of 348 migraineurs. Here, the effect of a low GI diet was non-inferior to pharmacological migraine prophylaxis [25].

Personalized nutrition represents an evolution of low GI approaches. In 2015, a large prospective study with more than 800 participants demonstrated that postprandial glucose responses (PPGR) to identical test meals are highly variable across individuals and influenced by anthropometry and the individual composition of the intestinal microbiome [26]. Berry et al. not only confirmed the strong interindividual effects of identical meals but additionally demonstrated that numerous other factors such as genetics, premeal physical activity, and sleep influence individual PPGR [27]. Together, these studies led to a paradigm shift in nutritional medicine. Not the food itself is considered the main determinant of the biological effect of nutrition, but individual factors such as microbiome, genetics, or lifestyle.

In our study, we explored whether personalized nutrition targeting the PPGR may influence migraine disease severity. We surveyed 238 migraine patients, who took part in a digital nutrition program that generates personalized dietary recommendations based on the analysis of individual continuous glucose measurements. A total of 84 individuals completed the retrospective digital survey. Using retrospective analysis, we were able to obtain first data indicating that a digital health application allowing a personalized low-glycemic nutrition has a potential prophylactic effect on migraine.

## 2. Materials and Methods

### 2.1. Digital Nutrition Program

As part of a digital nutrition program of Perfood GmbH (Lübeck, Germany), participants carry out a 10 to 14-day-long test phase in which they track their meals and physical activity via an app and eat defined test meals. During this period, the participants wear a glucose sensor for continuous glucose monitoring (CGM). At the end of the test phase, all participants receive a personalized low-glycemic nutrition (PLGN) report based on the individual measured data in relation to the meals consumed. For this purpose, the postprandial glucose curves are automatically evaluated using automated proprietary procedures. By comparing standardized test meals, a stratification into generally valid nutrition types is possible. These nutrition types include statements about frequently consumed carbohydrate-rich foods such as bread, cereals, pasta, potatoes, or the blood glucose-modulating influence of protein or fat. Thus, the nutrition types cover the majority of foods consumed in a typical diet of Western countries. This approach is complemented by comparing other users’ frequent meals in order to analyze daily meals with beneficial and detrimental effects on the individual PPGR. Daily diets were able to be modified by the users assuring low glycemic reactions. Of note, this approach is not a restrictive diet eliminating the complete group of carbohydrate-rich foods. On the contrary, personalization allows to merely reduce the specific foods responsible for unwanted high-glycemic reactions.

### 2.2. Data Collection

Data were collected via a retrospective survey using a digital questionnaire. Participation in the survey was voluntary. Patients were asked to participate by e-mail. All migraine patients who had previously participated in the digital nutrition program and therein had received a PLGN report between July 2018 and July 2020 were invited to participate in the survey. Four weeks was chosen as the minimum time period between receipt of the personalized report and the survey, as previous studies on the effects of dietary interventions on migraine symptoms have shown clinical effects after four-week interventions [28,29]. No further requirements were defined with respect to migraine severity or treatment regimen. All patients reported a physician-confirmed diagnosis; however, no written confirmation was obtained. Due to the retrospective nature, recording of daily headaches in a diary was not possible and, as a result, no classification using the International Classification of Headache Disorders (ICHD) was performed. The cohort is described in the results section.

The questionnaire was designed for the survey. The survey of the frequency of migraine days was guided by the guidelines of the International Headache Society for the implementation of clinical studies with migraine patients and focused on reported days with migraine headaches [30]. However, due to the retrospective method, no run-in baseline phase or the use of a daily headache diary could be implemented. Further migraine-specific questions were guided by the validated MIDAS and HIT-6 questionnaires. The MIDAS questionnaire is used to assess headache-related disability in migraine patients during the past three months [31,32,33]. The HIT-6 is validated for a period of 4 weeks and reflects the impact of headaches on daily life [34,35]. The questionnaire consisted of 4 items within the category “general condition”, 30 items within the category “migraine diagnosis and course of disease”, and 9 items within the category “dietary adherence and nutritional habits”.

### 2.3. Data Analysis and Statistics

All patients gave their consent to the general use of their anonymized data for scientific purposes. All data sets were anonymized and analyzed independently by the Institute of Medical Biometry and Statistics at the University of Lübeck. The study was approved by the ethics committee of the University of Lübeck (AZ 20-4015). The data of all survey participants were included and relevant endpoints before program participation (TP0) and after program participation (TP1, time of survey) were compared. Endpoints were changes in the number of migraine days per 28 days, duration of attacks in hours, pain level (on a scale from 1–10), intake of pain medication in days per 28 days, absenteeism (defined as number of days with absence from work or school/university due to migraine symptoms per 28 days), and presenteeism (defined as the number of days with reduced performance because of migraine complaints per 28 days). To assess the intraindividual change from TP0 to TP1, paired t-tests were used, where *p*-values were obtained from permutation (with 99.999 runs) to allow for deviations from the normal distribution. To account for the multiple testing of six endpoints, the significance level was adjusted according to Bonferroni–Holm. The results are interpreted as statistically significant if the p-value is lower than the respective adjusted significance level. Given that we have a single-arm study with intraindividual comparisons, we did not include any further adjustments.

### 2.4. Subgroup Analyses

For further insight, participants were subsequently divided into two groups according to their reported adherence to the dietary recommendations. These groups will be referred to as “adherent” and “non-adherent” in the following, with the “adherent” group including those participants who reported adherence to the dietary recommendations since receiving the report. The “non-adherent” group thus comprised all participants not reporting adherence since receiving the report. Individuals who did not answer this item were coded as “non-adherent”. Based on these categories, exploratory analyses were performed to simultaneously estimate the associations of time (TP0 and TP1) and adherence (“adherent” and “non-adherent”) with migraine severity. A rank-based non-parametric analysis of variance for longitudinal data (ANOVA-type statistics) was utilized [36]. Specifically, the test for the time factor indicates before-and-after differences, and the test for the interaction of time and group factor indicates possible differences between the “adherent” and “non-adherent” group in terms of the before-and-after change. “Relative treatment effects” (RTE) were used as effect size. These can be interpreted as follows: if one individual is randomly drawn from the adherent group and one from the non-adherent group, the RTE gives the probability that the individual in the adherent group has a greater before-and-after change than the individual in the non-adherent group. A probability value that is higher or lower than 0.50 indicates a positive (for >0.50) or negative (for <0.50) association of the adherence (to the recommendations) with the before-and-after change. Because of the exploratory nature of this analysis, no correction was made for multiple testing, and *p*-values are interpreted descriptively.

Furthermore, participants who reported having a migraine more than 1 day per month at baseline were classified as having “regular” migraines. Patients with 1 migraine day per month at baseline were classified to have a “rare” form of migraine for the sake of additional subgroup analyses. This approach followed the rationale that median migraine days are reported to be 1 migraine day per month in Germany [37]. Just 3 subjects reported to have more than 14 days per month of migraine and can be suspected to have a chronic migraine based on the classification of the International Headache Society [38]. However, due to the low number and the proof-of-concept nature of this study, these 3 subjects were not eliminated from the analyses. Subsequently, the non-parametric analysis of variance was extended by including an additional factor of migraine frequency (regular episodic migraine and rare episodic migraine) with the estimation of RTE. Again, *p*-values are interpreted descriptively.

## 3. Results

### 3.1. Cohort Description

**Figure 1:**
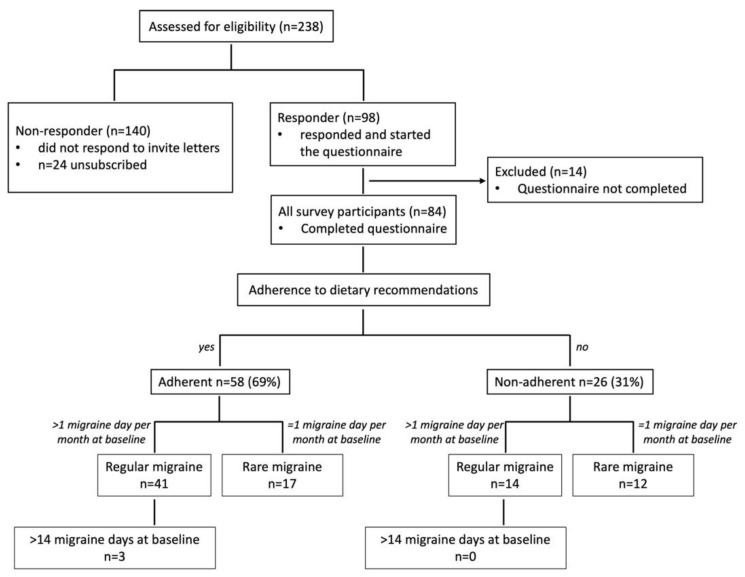
Study recruitment and classification of participants into (sub)groups. All patients who had been assessed for eligibility (*n* = 238 migraine patients) were contacted and asked to take part in the online survey. A total of 24 patients unsubscribed from the mailing list or were not reachable, 98 responded and started the questionnaire (response rate 45.8%), and 84 completed the questionnaire. Participants were categorized according to their adherence to dietary recommendations. These groups were further subdivided into migraine patients who reported a regular form of migraine (>1 day with migraine per month at baseline) or a rare form of migraine (=1 day migraine per month). A total of 3 subjects within the adherent group reported more than 14 days per month with migraine.

Study recruitment and classification of participants into (sub)groups are illustrated in the flowchart in Figure 1. All 84 survey participants were classified based on reported days per month with migraine at baseline and on reported adherence to the dietary recommendation between program participation and the survey. Patients were not further classified in episodic and chronic migraine because of a lack of decisive data from a headache diary. Patients were asked for days with typical migraine headaches but due to the retrospective analysis, it is not possible to verify using the ICHD criteria.

Table 1 shows the baseline descriptive data of all 84 patients, who could be included in the following analyses. The mean age was 42.6 years (SD 10.7) and 90.5% were female patients. Mean BMI was 27.4 kg/m^2^ (SD 6.4). The mean time since the receipt of the PLGN report was 52.4 weeks (SD 28.7). The mean time since migraine diagnosis was 19.7 years (SD 11.3). Basic demographic data were available for the complete study population, so the responding participants were compared with the other program participants (*n* = 140, data not shown). In this analysis, the mean age of non-responders was 42.9 years (SD 11.6) and 92.7% were female patients. Mean BMI was 26.2 kg/m^2^ (SD 4.84) and the mean time since the receipt of the PLGN report was 63.2 weeks (SD 28.0). Collectively, this comparison indicates that the survey participants were representative of all program participants.

Survey participants were asked about medical care; a total of 57.1% of the survey participants stated that they received frequent medical care from a GP or neurologist. However, less than 10% were on prophylactic medication. A total of 83.3% stated that they usually intake analgesic medication and nearly 30% usually intake triptans when having a migraine attack. Mean monthly migraine days were 3.5 (SD 4.3) with a mean pain level of 7.2/10 (SD 1.5) and a mean duration of 21.6 h (SD 23.0). Presenteeism was reported on a mean of 3.2 (SD 3.9) days per month and absenteeism on a mean of 1.0 day (SD 1.6) per month.

The baseline descriptions of the subgroups based on adherence and baseline frequency of migraine can be found in the Supplementary Materials, Tables S1–S6. In summary, no major difference at baseline was observed; however, no statistical comparisons were performed at baseline.

### 3.2. Migraine Symptoms after Receipt of the PLGN Report

**Figure 2:**
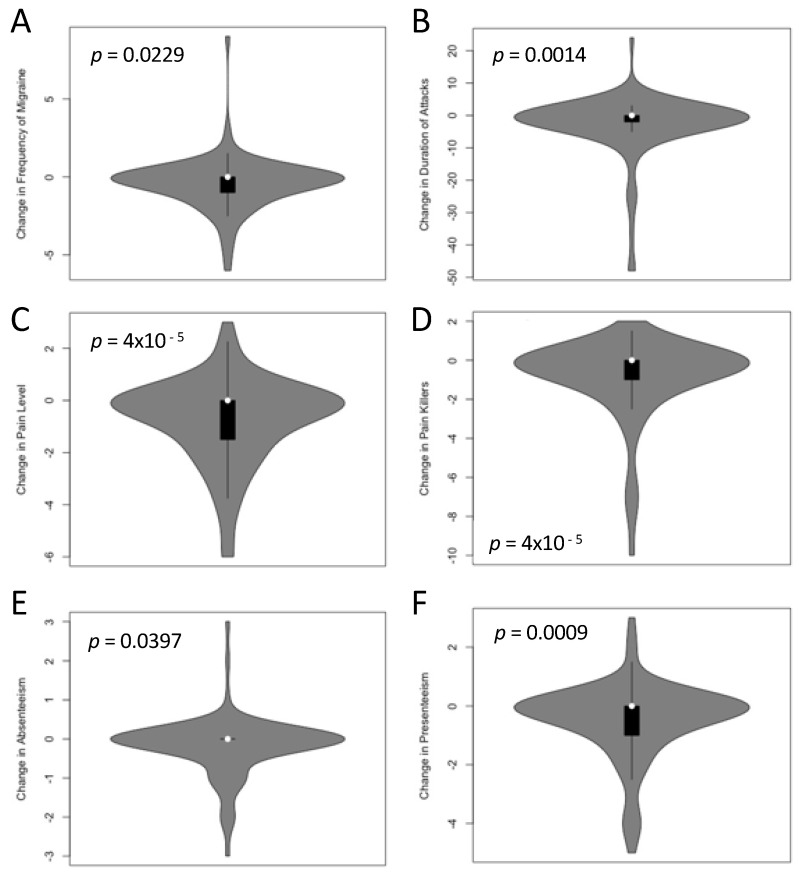
Change of migraine symptoms at TP0 (baseline, before participation in the program) and TP1 (after receiving the personalized, low-glycemic nutrition report). Monthly migraine frequency in days per 28 days (**A**), duration of the attack in hours (**B**), pain level on a scale of 1–10 (**C**), monthly intake of medication in days per 28 days (**D**), absenteeism (**E**), and presenteeism (**F**) in days per 28 days were significantly lower at TP1 than at TP0 (all *p* < 0.05 in paired permutation t-tests after adjustment for multiple testing). The individual *p*-values are indicated in the figures.

Table 2 describes migraine symptoms at the time of the survey (TP1, i.e., after receiving the PLGN report) and the intraindividual changes between TP0 (baseline) and TP1. The patient-relevant symptoms frequency of migraine, pain level, duration of attacks, painkiller intake, absenteeism, and presenteeism were assessed; all these symptoms were significantly lower after the nutrition program at the time of the survey. Figure 2 displays the intraindividual comparisons with *p*-values from paired permutation t-tests.

### 3.3. Impact of Adherence to the PLGN Report

For further analyses, the patients were categorized based on their adherence to the personalized, low-glycemic diet recommendations (Figure 1). Table 3 shows the distribution of the endpoints in the adherent group (*n* = 58) and Table 4 in the non-adherent group before and after program participation. Of note, a reduction in symptoms was more dominant in the adherent than the non-adherent group.

**Figure 3:**
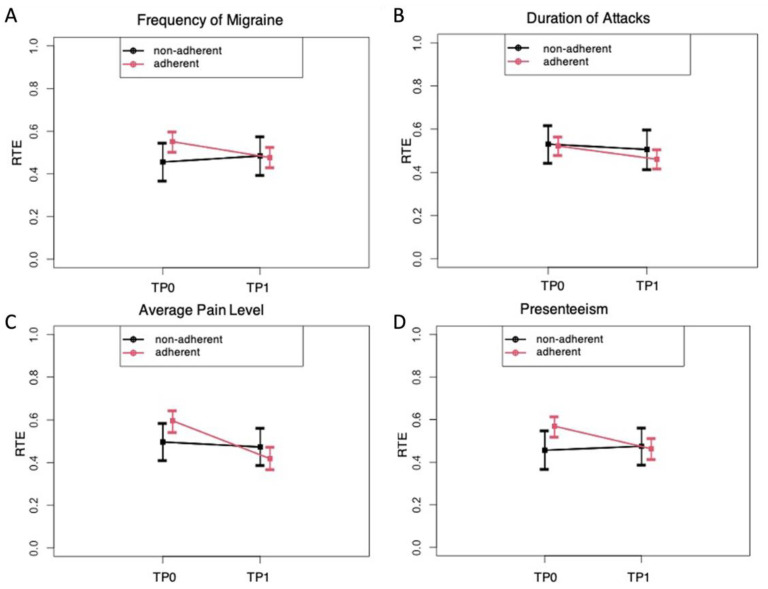
“Relative treatment effects” (RTE) on migraine with 95% confidence intervals depending on adherence to the personalized low-glycemic nutrition report (adherent versus non-adherent). RTE of adherence on the parameters of migraine frequency (**A**), duration of attack (**B**), pain level (**C**), and presenteeism (**D**) at the evaluation times TP0 (before) and TP1 (after). Rank-based non-parametric analyses of variance for longitudinal data were carried out (ANOVA-type statistics). (**A**) The RTE on the frequency of migraines indicates an association of a decrease over time in the adherent but not in the non-adherent group (descriptive p for time × group interaction = 0.033). (**B**) The RTE on the duration of the attack indicates a stronger association of a decrease in the adherent than in the non-adherent group (descriptive p for time × group interaction = 0.069). (**C**) The RTE on average pain levels indicates a stronger association of a decrease in the adherent than in the non-adherent group (descriptive p for time × group interaction = 0.008). (**D**) The RTE on presenteeism indicates an association of a decrease in the adherent and not in the non-adherent group (descriptive p for time × group interaction = 0.0004).

For further statistical analyses of the adherent and the non-adherent groups, the “relative treatment effects” (RTEs) of the adherent versus non-adherent group on the change of symptoms from baseline (TP0) to after receiving the PLGN report (TP1) were calculated for migraine frequency, attack duration, pain intensity, and presenteeism as the most interesting parameters (Figure 3). The RTEs showed that adherence as compared with non-adherence was associated with a greater reduction in the frequency of migraine (Figure 3A), pain intensity (Figure 3C), and presenteeism (Figure 3D). Body weight did not decrease over time in all survey participants (descriptive *p* = 0.095), and no interaction of time and group factor was observed, indicating that the effects on migraine symptoms were not related to weight loss in the adherent compared with the non-adherent group (data not shown).

### 3.4. Impact of Baseline Migraine Frequency on Migraine Symptoms

For the following analyses, the adherent and non-adherent patients were further divided into “rare” and “regular” migraine based on baseline migraine frequency with “regular” classified as more than 1 monthly migraine day. In line with the analyses before, the distribution is described for patients with “rare” and “regular” migraine for the adherent and non-adherent groups separately (Table 5 and Table 6 show adherent patients; non-adherent patients are stated in Supplementary Materials, Tables S7 and S8).

Focusing on the patients with regular migraine at baseline enlarged the reduction in migraine symptoms when comparing adherent vs. non-adherent groups. Patients who adhered to the nutrition recommendations of their PLGN report experienced a mean change in frequency of migraine of −1.3 days (SD 1.5), in the pain level of −1.1 (SD 1.6), in the duration of attacks of −5.7 (SD 12.5), in painkiller intake of −1.4 days (2.2), in absenteeism of −0.5 days (SD 0.8), and in presenteeism of −1.0 days (SD 1.4) (Table 5). Patients not adhering to the recommendations experienced nearly no change (Table 6).

**Figure 4:**
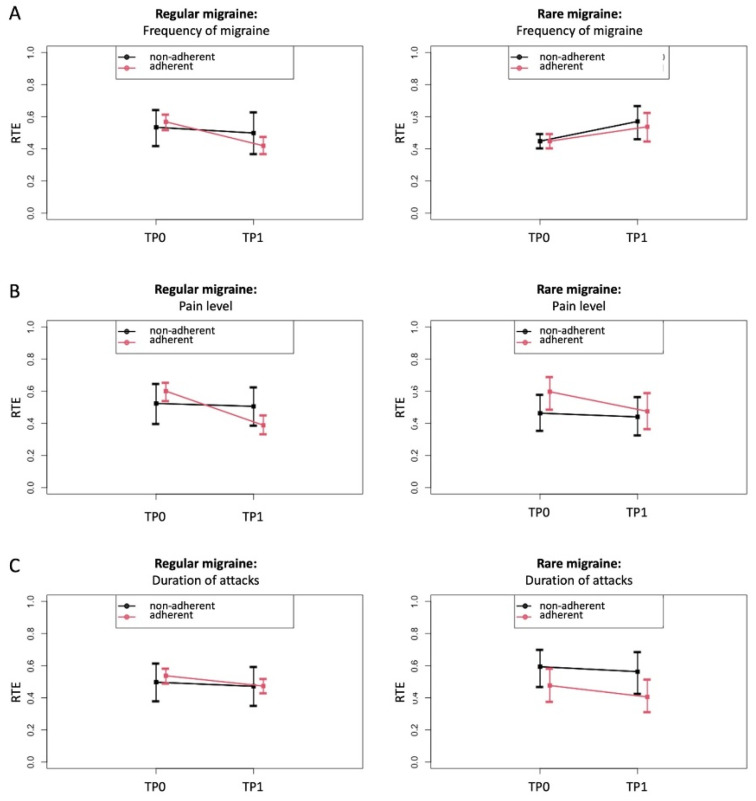
Relative treatment effects (RTE) on migraine with 95% confidence intervals depending on adherence to the personalized low-glycemic diet recommendations (adherent versus non-adherent), shown separately for patients with regular and rare migraine migraines. RTE of adherence on the parameters migraine frequency (**A**), pain level (**B**), and duration of attack (**C**) at TP0 (before) and TP1 (after) for regular migraine (left images) and rare migraine (right images). This was done separately for patients with regular and rare migraine. Rank-based non-parametric variance analyses were carried out for longitudinal data (ANOVA-type statistics). (**A**) RTE on migraine frequency indicates an association with a decrease in the adherent group for regular migraine (descriptive p for time × group interaction = 0.0029) and not for rare migraine (descriptive p for time × group interaction = 0.7099). (**B**) RTE on the duration of the attack indicates a stronger association with a decrease in the adherent than in the non-adherent group (descriptive p for time × group interaction = 0.0194 for regular migraine and descriptive p for time × group interaction = 0.4474 for rare migraine). (**C**) RTE on pain level indicates an association with a decrease in both groups but with a stronger effect in adherent patients (descriptive p for time × group interaction = 0.109 for regular migraine and descriptive p for time × group interaction = 0.2825 for rare migraine).

To further explore these effects, the RTEs of the adherent versus non-adherent group on the changes from baseline (TP0) to after receiving the PLGN report (TP1) were estimated for migraine frequency, attack duration, and pain intensity as relevant parameters. The RTEs showed that adherence compared with non-adherence was associated with a lower frequency of migraine (Figure 4A) and pain intensity (Figure 4B) only in “regular” migraine but not in “rare” migraine. No clear time or group-specific effects were seen on the duration of migraine attacks (Figure 4C) or other migraine symptoms (data not shown).

## 4. Discussion

The objective of the present study was to produce the first proof-of-concept of whether a personalized low-glycemic nutritional intervention as a digital therapy can potentially exert clinically relevant effects in the prophylaxis of migraine attacks. Indeed, data from the present study indicate that an individually tailored low-glycemic dietary intervention may be effective in the prophylaxis of migraine.

A complete cohort of 84 patients who reported having a physician-confirmed migraine, was set up. The cohort was heterogeneous with respect to baseline migraine frequency (from a minimum of 1 migraine day per month to a maximum of 28 days per month) and related migraine symptoms. The cohort was mostly female, had concomitant tension-type headaches in 56%, frequently received antimigraine medical care in 57.1%, frequently used acute medication such as painkillers or triptans, and less frequently used prophylactic medications. A total of 29 patients (34.5%) reported having one migraine day per month at baseline classified as “rare” migraine in order to distinguish it from the remaining 55 patients with “regular” migraine. Of these, three subjects reported more than 14 migraine days per month at baseline and could be suspected to have chronic migraine. However, due to the lack of data collected in a headache diary and the retrospective nature, this issue was not further considered for the analyses performed. Taken together, the complete cohort can be considered representative for patients with migraine. However, future research should collect data with more detailed information about the clear characteristics of the reported headaches.

An analysis of reported clinically relevant symptoms before and after receiving the PLGN report, such as attack frequency, pain level, duration of attacks, the need for painkiller intake, and days with absenteeism or presenteeism, were significantly lower after taking part in this nutritional program as compared with the situation before. This highlights the potential therapeutic effect of this nutritional approach in migraine prophylaxis. However, these data need confirmation with a prospective data collection and additional measures addressing potential confounding factors.

To further evaluate whether these changes may depend on the fact that 58 patients reported to daily follow the personalized nutrition recommendation (“adherent subgroup”) and 26 reported following the recommendation less than once a day (“non-adherent subgroup”), we separately analyzed the changes for adherent and non-adherent patients. In fact, the changes were clearly predominant in the adherent subgroup. Specifically, our non-parametric analysis of variance indicated that the intraindividual changes were more prominent in the adherent group. In further subgroup analyses of patients with regular migraine, those who were adherent to the personalized recommendations reported a mean reduction in migraine frequency of 33%. According to expert assessment, this alone can be classified as clinically relevant [39]. In 38% of patients with regular migraines, the frequency of migraines even improved by at least 50% (i.e., response rate). Placebo-controlled studies on the effect of the new migraine-specific CGRP antibodies in migraine prophylaxis showed a 36% to 43% response rate [40,41,42,43]. This indicates the potential effectiveness of the PLGN report in the range of drug effects. In addition, this finding is in line with previous reports about the positive impact of diets that stabilize glycemic reactions on migraine symptoms [24,25,29].

It has to be appreciated that both factors, adherence to the recommendations and change in migraine severity, were assessed in a retrospect manner; so it cannot be ruled out that both observations may interact. Accordingly, this analysis does not allow for confirmatory conclusions about the causative role of the nutrition intervention; however, it can be considered as the first proof-of-concept data that following personalized low-glycemic nutritional recommendation has the potential to reduce migraine severity.

At this point, it remains uncertain by which exact mechanisms a personalized low-glycemic diet may reduce migraine symptoms. One possible mechanism may be via an increased insulin secretion that occurs in association with high PPGR. After ingestion of rapidly absorbable carbohydrates, blood glucose levels frequently drop to levels below pre-prandial blood glucose levels. This phenomenon is usually explained by excessive insulin secretion in the sense of a complex physiological counterregulatory response [44]. Part of this compensatory response could be an increased CGRP secretion. CGRP has an effect on glucose levels and is capable of inducing relative hyperglycemia [45]. Vice versa, CGRP levels are elevated in patients during a migraine attack [46], which is discussed in the literature as a consequence of a presumed migraine-specific central nervous energy deficit after an excess energy usage before the attack, among other factors [23]. However, it remains speculative if the hyperinsulinemia characteristically observed in migraine patients represents a counterregulatory response to chronic CGRP elevation [47,48] or might be even the origin of this phenomenon. The consequence of following a low-glycemic food is a reduction in PPGR and an improvement of systemic insulin sensitivity.

It is debated whether and how diabetes mellitus and migraine may be interrelated [49]. Epidemiological data suggest that diabetes is less frequent in migraine patients and vice versa [50]. A diverging regulation of CGRP is one approach to an explanation [50]; however, underlying mechanisms are still elusive.

In addition, a low-glycemic diet potentially has an impact on the central nervous system [48]. As an alternate hypothesis, a low-glycemic approach may favor economization of the central nervous energy supply, thereby counteracting the central nervous energy deficit suspected in migraine [23]. Furthermore, a reduction in oxidative stress levels could also be an explaining mechanism [51,52,53].

A low-glycemic approach may be beneficial in various diseases [54,55]; however, inconsistent effects are reported in the prevention and management of chronic diseases [56,57,58]. This may be due to the high interindividual variance of the PPGR [26,27]. In fact, it is increasingly appreciated that effective personalization strategies are needed to tailor dietary recommendations to the individual’s metabolism. The obvious advantage over generalized low-glycemic recommendations is that personalized nutrition approaches based on objectively measured data—such as CGM data—take into account individual metabolism and consequently are more precise than general dietary recommendations [27].

## 5. Limitations

Of note, the data should be interpreted cautiously since several limitations arise from the retrospective study design.

First, the patients were asked to recall their migraine symptoms from the time before participating in the program. The mean time since the receipt of the personalized, low-glycemic nutrition report was 56.4 months with a minimum of 5.7 months and a maximum of 118 months. This leads to a potential recall bias. However, it is described that, when recording migraine days retrospectively, patients can tend to underestimate baseline numbers of headaches in periods that are longer ago [59]. Therefore, it can also be speculated that the frequency of migraine headaches was underestimated at the baseline rather than at the time of the survey and that the effect described could actually be even higher. However, no statistical relationship was detected between the individual observation time (time between baseline TP0 and survey TP1) and the specified baseline disease severity or the individual change in migraine days.

Second, the patients reported a physician-confirmed diagnosis, but no written confirmation was obtained, which could lead to a misclassification bias. In addition, the missing headache diary does not allow checking for ICHD criteria.

Third, no control measure for other migraine interventions such as other prophylactic drugs or non-pharmacological interventions was taken. In addition, the study does not involve a control group with a different or no treatment. This leads to uncertainty that the change in migraine severity could be induced by other factors than the nutritional intervention.

Forth, not all approached patients took part in the survey, the response rate was 45.8%. No data about the baseline disease severity of the course of the disease could be analyzed from the non-responding patients leading to a potential selection bias. In addition, patients responding to the survey request may have their own ideas about the involvement of nutrition and may tend to not objectively report their symptoms after the intervention (reporting bias).

Fifth, it is unknown why the patients took part in the nutrition program in the first place as it was not specifically advertised as a nutritional program for migraine prophylaxis. The reason for participation could also interfere with the study results. On the one hand, not knowing about a potential effect could reduce a confounding “placebo effect”, and on the other hand, the motivation to adhere or not adhere to the dietary recommendation could be influenced by factors unconnected to the disease.

More open questions arise from this study; e.g., the question remains whether the avoidance of an individually hyperglycemic postprandial glucose response is causally related to the observed effects or whether other factors, such as the sole use of a nutrition app, also have an influence on the frequency and severity of migraine patients. The fact that those participants who did not follow the dietary recommendations did not report improvement in clinical migraine symptoms supports the view of a causal relation.

## 6. Conclusions

Despite several limitations, we could present noteworthy proof-of-concept data for a digitally enabled personalized nutrition therapy in patients with episodic migraine. As a bottom line, a personalized low-glycemic diet was associated with a clinically significant improvement in patients with more than 1 monthly migraine day. This makes this nutrition program a potential therapy candidate for patients with episodic migraine to overcome obstacles of pharmacological approaches. In addition to elucidating the underlying principles of action, clinical efficacy now needs to be demonstrated in randomized controlled trials.

## Figures and Tables

**Table 1:** Baseline description of all survey participants.

Baseline Description of All Survey Participants			
	Number	Percentage	
Sample size	84	100%	
Female participants	76	90.5%	
Participants with frequent medical care with GP/neurologist	48	57.1%	
Participants with concomitant tension-type headache	47	56,0%	
Participants with prophylactic medication (beta blocker, amitriptylin, flunarizin, topiramat, valproate)	7	8.3%	
Participants with CGRP antibody treatment	1	1.2%	
Participants with Magnesium intake	13	15.5%	
Participants with painkiller intake (NSAR, metamizol, paracetamol)	70	83.3%	
Participants with intake of triptanes	25	29.8%	
	mean ± SD	median (IQR)	min; max
Age [years]	42.6 ± 10.7	45.0 (15.5)	18; 62
Body Mass Index [kg/m2]	27.4 ± 6.4	26.3 (6.9)	18.0; 50.6
Time of diagnosed migraine disease [years]	19.7 ± 11.3	19.0 (20.0)	1; 42
Time between program participation and survey [weeks]	52.4 ± 28.7	58.7 (43.4)	5.7; 118.0
Frequency of migraine [days per month]	3.5 ± 4.3	2.0 (2.5)	1; 28
Average pain level [1–10]	7.2 ± 1.5	7.0 (2.0)	3; 10
Duration of migraine attacks [hours]	21.6 ± 23.0	11.0 (23.5)	1; 96
painkiller intake [days per month]	3.4 ± 4.0	2.0 (3.0)	0; 20
Absenteeism [days per month]	1.0 ± 1.6	0.0 (1.0)	0; 10
Presenteeism [days per month]	3.1 ± 3.9	2.0 (3.0)	0; 28

**Table 2:** Description of symptoms after program participation for all patients.

Sample Size *n* = 84	Mean ± SD	Median (IQR)	Min; Max
Frequency of migraine [days per month]	3.0 ± 4.0	2.0 (2.0)	0; 28
change from TP0 to TP1	−0.4 ± 1.7	0.0 (1.0)	−6; 9
Pain level [1–10]	6.4 ± 1.8	7.0 (3.0)	1; 10
change from TP0 to TP1	−0.8 ± 1.6	0.0 (1.5)	-6; 3
Duration of migraine attacks [hours]	18.7 ± 20.1	8.0 (20.0)	1; 72
change from TP0 to TP1	−3.1 ± 9.9	0.0 (2.0)	−48; 24
Painkiller intake [days per month]	2.5 ± 3.1	1.0 (1.0)	0; 15
change from TP0 to TP1	−0.9 ± 2.1	0.0 (1.0)	−10; 2
Absenteeism [days per month]	0.8 ± 1.5	0.0 (1.0)	0; 10
change from TP0 to TP1	−0.2 ± 0.8	0.0 (0.0)	−3; 3
Presenteeism [days per month]	2.6 ± 3.8	1.0 (2.0)	0; 28
change from TP0 to TP1	−0.5 ± 1.3	0.0 (1.0)	−5; 3

All survey participants (*n* = 84) reported migraine symptoms at the time of survey (i.e., after program participation, TP1) and recalled migraine symptoms from the the time before programm participation (TP0). Reported symptoms at TP1 and the change of symptoms from TP0 to TP1 are presented. Data presented as mean, standard deviation (SD), median, interquartile range (IQR), minimum (min), and maximum (max).).

**Table 3:** Description of symptoms after program participation for all adherent patients.

Sample Size *n* = 58	Mean ± SD	Median (IQR)	Min; Max
Frequency of migraine [days per month]	3.0 ± 4.3	2.0 (2.0)	0; 28
change from TP0 to TP1	−0.8 ± 1.5	0.0 (2.0)	−6; 3
Pain level [1–10]	6.3 ± 1.8	6.0 (3.0)	1; 10
change from TP0 to TP1	−1.0 ± 1.8	0.0 (2.0)	−6; 2
Duration of migraine attacks [hours]	18.4 ± 20.9	8.0 (20.0)	1; 72
change from TP0 to TP1	−4.2 ± 10.7	0.0 (2.0)	−48; 4
Painkiller intake [days per month]	2.6 ± 3.2	1.0 (1.3)	0; 15
change from TP0 to TP1	−1.0 ± 2.1	0.0 (1.0)	−8; 2
Absenteeism [days per month]	0.7 ± 1.1	0.0 (1.0)	0; 5
change from TP0 to TP1	−0.3 ± 0.8	0.0 (0.0)	−3; 2
Presenteeism [days per month]	2.7 ± 4.3	1.0 (2.0)	0; 28
change from TP0 to TP1	−0.8 ± 1.4	0.0 (0.0)	−5; 3

All survey participants with adherence to the nutritional recommendations (*n* = 58) reported migraine symptoms at the time of survey (i.e., after program participations, TP1) and recalled migraine symptoms from the the time before programm participation (TP0). Reported symptoms at TP1 and the change of symptoms from TP0 to TP1 are presented. Data presented as mean, standard deviation (SD), median, interquartile range (IQR), minimum (min), and maximum (max).

**Table 4:** Description of symptoms after program participation for all non-adherent patients.

Sample Size *n* = 26	Mean ± SD	Median (IQR)	Min; Max
Frequency of migraine [days per month]	3.0 ± 3.1	2.0 (2.0)	1; 10
change from TP0 to TP1	0.3 ± 1.8	0.0 (0.0)	−1; 9
Pain level [1–10]	6.7 ± 1.6	7.0 (1.8)	3; 10
change from TP0 to TP1	−0.1 ± 1.1	0.0 (0.0)	−2; 3
Duration of migraine attacks [hours]	19.4 ± 18.6	11.0 (23.0)	2; 72
change from TP0 to TP1	−0.8 ± 7.5	0.0 (0.0)	−26; 24
Painkiller intake [days per month]	2.3 ± 3.1	1.0 (1.0)	0; 10
change from TP0 to TP1	−0.6 ± 2.1	0.0 (0.0)	−10; 0
Absenteeism [days per month]	1.0 ± 2.1	0.0 (1.0)	0; 10
change from TP0 to TP1	0.1 ± 0.6	0.0 (0.0)	−1; 3
Presenteeism [days per month]	2.5 ± 2.8	1.5 (2.0)	0; 10
change from TP0 to TP1	0.1 ± 0.5	0.0 (0.0)	−1; 2

All survey participants without adherence to the nutritional recommendations (*n* = 26) reported migraine symptoms at the time of survey (i.e., after program participations, TP1) and recalled migraine symptoms from the the time before programm participation (TP0). Reported symptoms at TP1 and the change of symptoms from TP0 to TP1 are presented. Data presented as mean, standard deviation (SD), median, interquartile range (IQR), minimum (min), and maximum (max).

**Table 5:** Description of symptoms after program participation for adherent patients with regular migraine.

Sample Size *n* = 40	Mean ± SD	Median (IQR)	Min; Max
Frequency of migraine [days per month]	3.8 ± 5.0	2.0 (3.0)	1; 28
change from TP0 to TP1	−1.3 ± 1.5	−1.0 (2.0)	−6; 0
Pain level [1–10]	6.3 ± 1.6	6.0 (2.0)	3; 10
change from TP0 to TP1	−1.1 ± 1.6	−0.5 (2.0)	−6; 1
Duration of migraine attacks [hours]	24.1 ± 22.7	20.0 (43.3)	1; 72
change from TP0 to TP1	−5.7 ± 12.5	0.0 (4.0)	−48; 0
painkiller intake [days per month]	3.2 ± 3.6	2.0 (3.0)	0; 15
change from TP0 to TP1	−1.4 ± 2.2	0.0 (2.0)	−8; 0
Absenteeism [days per month]	0.8 ± 1.3	0.0 (1.0)	0; 5
change from TP0 to TP1	−0.5 ± 0.8	0.0 (1.0)	−3; 0
Presenteeism [days per month]	3.3 ± 4.9	2.0 (2.3)	0; 28
change from TP0 to TP1	−1.0 ± 1.4	0.0 (2.0)	−5; 0

All survey participants with adherence to the nutritional recommendations and frequent migraine at baseline (>1 migraine day per month) (*n* = 40) reported migraine symptoms at the time of survey (i.e., after program participations, TP1) and recalled migraine symptoms from the the time before programm participation (TP0). Reported symptoms at TP1 and the change of symptoms from TP0 to TP1 are presented. Data presented as mean, standard deviation (SD), median, interquartile range (IQR), minimum (min), and maximum (max).

**Table 6:** Description of symptoms after program participation for non-adherent patients with regular migraine.

Sample Size *n* = 14	Mean ± SD	Median (IQR)	Min; Max
Frequency of migraine [days per month]	4.0 ± 3.4	2.5 (2.0)	1; 10
change from TP0 to TP1	−0.1 ± 0.4	0.0 (0.0)	−1; 0
Pain level [1–10]	7.0 ± 1.4	7.0 (1.8)	5; 10
change from TP0 to TP1	−0.1 ± 1.2	0.0 (0.0)	−2; 3
Duration of migraine attacks [hours]	20.3 ± 15.4	24.0 (20.5)	2; 48
change from TP0 to TP1	−1.2 ± 3.5	0.0 (0.0)	−12; 2
painkiller intake [days per month]	3.6 ± 3.6	2.0 (2.5)	0; 10
change from TP0 to TP1	−0.3 ± 0.8	0.0 (0.0)	−2; 0
Absenteeism [days per month]	1.2 ± 2.6	0.5 (1.0)	0; 10
change from TP0 to TP1	0.0 ± 0.0	0.0 (0.0)	0; 0
Presenteeism [days per month]	3.0 ± 3.2	2.0 (2.0)	0; 10
change from TP0 to TP1	0.0 ± 0.0	0.0 (0.0)	0; 0

All survey participants without adherence to the nutritional recommendations and frequent migraine at baseline (>1 migraine day per month) (*n* = 14) reported migraine symptoms at the time of survey (i.e., after program participations, TP1) and recalled migraine symptoms from the the time before programm participation (TP0). Reported symptoms at TP1 and the change of symptoms from TP0 to TP1 are presented. Data presented as mean, standard deviation (SD), median, interquartile range (IQR), minimum (min), and maximum (max).

## Data Availability

The datasets used and/or analyzed during the current study are available from the corresponding author on reasonable request.

## Supplementary Materials

The following are available online at https://www.mdpi.com/article/10.3390/jcm11041117/s1: Table S1: Baseline description of all non-adherent survey participants, Table S2: Baseline description of all adherent survey participants, Table S3: Baseline description of all non-adherent survey participants with regular migraine, Table S4: Baseline description of all adherent survey participants with regular migraine, Table S5: Baseline description of all non-adherent survey participants with rare migraine, Table S6: Baseline description of all adherent survey participants with rare migraine, Table S7: Description of symptoms after program participation for non-adherent patients with rare migraine, Table S8: Description of symptoms after program participation for adherent patients with rare migraine.
