# Supplementary material for: A Digital Health Application Allowing a Personalized Low-Glycemic Nutrition for the Prophylaxis of Migraine: Proof-of-Concept Data from a Retrospective Cohort Study
Source: J Clin Med. 2022 Feb 20;11(4):1117. doi: 10.3390/jcm11041117 (PMC8878080; doi:10.3390/jcm11041117)
Supplement: Supplementary file 1 [file jcm-11-01117-s001.zip › jcm-1585101-supplementary.pdf]

Supplementary Tables

Table S1. Baseline description of all non-adherent survey participants.

**Baseline description of non-adherent survey participants**			
	number	percentage	
Sample size	26	100%	
Female participants	20	76.9%	
Participants with frequent medical care with GP/neurologist	15	57.7%	
Participants with concomitant tension-type headache	12	46.2%	
Participants with prophylactic medication (beta blocker, amitriptylin, flunarizin, topiramat, valproate)	2	7.7%	
Participants with CGRP antibody treatment	0	0.0%	
Participants with Magnesium intake	1	3.8%	
Participants with painkiller intake (NSAR, metamizol, paracetamol)	22	84.6%	
Participants with intake of triptanes	7	26.9%	
	mean ± SD	median (IQR)	min; max
Age [years]	40.9 ± 10.7	41.0 (18.0)	18; 58
Body Mass Index [kg/m2]	25.8 ± 7.0	24.1 (4.3)	18.0; 50.6
Time of diagnosed migraine disease [years]	17.4 ± 10.9	15.5 (17.0)	3; 38
Time between program participation and survey [weeks]	57.9 ± 29.9	57.5 (41.3)	8.7; 118.0
Frequency of migraine [days per month]	2.6 ± 2.8	2.0 (1.8)	1; 10
Average pain level [1-10]	6.8 ± 1.5	7.0 (2.0)	4; 10
Duration of migraine attacks [hours]	20.2 ± 18.2	11.0 (24.5)	2; 62
painkiller intake [days per month]	2.9 ± 3.3	2.0 (2.0)	0; 16
Absenteeism [days per month]	0.9 ± 2.0	0.0 (1.0)	0; 10

Presenteeism [days per month]	2.4 ± 2.9	1.0 (2.0)	0; 10

Table S2. Baseline description of all adherent survey participants.

**Baseline description of adherent survey participants**			
	number	percentage	
Sample size	58	100%	
Female participants	56	96.6%	
Participants with frequent medical care with GP/neurologist	33	56.9%	
Participants with concomitant tension-type headache	35	60.3%	
Participants with prophylactic medication (beta blocker, amitriptylin, flunarizin, topiramat, valproate)	5	8.6%	
Participants with CGRP antibody treatment	1	1.7%	
Participants with Magnesium intake	12	20.7%	
Participants with painkiller intake (NSAR, metamizol, paracetamol)	48	82.8%	
Participants with intake of triptanes	18	31.0%	
	mean ± SD	median (IQR)	min; max
Age [years]	43.3 ± 10.8	46.5 (15.8)	20; 62
Body Mass Index [kg/m2]	28.1 ± 6.0	27.7 (6.7)	18.6; 43.3
Time of diagnosed migraine disease [years]	20.8 ± 11.4	20.0 (20.0)	1; 42
Time between program participation and survey [weeks]	55.7 ± 28.4	58.7 (43.6)	5.7; 114.9
Frequency of migraine [days per month]	3.8 ± 4.8	2.0 (3.0)	1; 28
Average pain level [1-10]	7.3 ± 1.5	7.0 (1.0)	3; 10
Duration of migraine attacks [hours]	22.2 ± 25.0	11.0 (21.3)	1; 96
painkiller intake [days per month]	3.6 ± 4.3	2.0 (3.3)	0; 20

Absenteeism [days per month]	1.0 ± 1.4	0.0 (2.0)	0; 5
Presenteeism [days per month]	3.4 ± 4.2	2.0 (3.0)	0; 28

Table S3. Baseline description of all non-adherent survey participants with regular migraine.

**Baseline description of non-adherent survey participants with regular migraine**			
	number	percentage	
Sample size	14	100%	
Female participants	12	85.7%	
Participants with frequent medical care with GP/neurologist	8	57.1%	
Participants with concomitant tension-type headache	7	50.0%	
Participants with prophylactic medication (beta blocker, amitriptylin, flunarizin, topiramat, valproate)	1	7.1%	
Participants with CGRP antibody treatment	0	0.0%	
Participants with Magnesium intake	1	7.1%	
Participants with painkiller intake (NSAR, metamizol, paracetamol)	11	78.6%	
Participants with intake of triptanes	4	28.6%	
	mean ± SD	median (IQR)	min; max
Age [years]	41.7 ± 11.4	43.0 (16.3)	18; 55
Body Mass Index [kg/m2]	24.5 ± 3.9	24.5 (4.2)	18.0; 31.1
Time of diagnosed migraine disease [years]	20.3 ± 12.1	19.0 (20.3)	3; 38
Time between program participation and survey [weeks]	57.1 ± 34.2	55.8 (48.3)	8.7; 118.0
Frequency of migraine [days per month]	4.1 ± 3.3	2.5 (2.0)	2; 10
Average pain level [1-10]	7.1 ± 1.4	7.0 (2.0)	5; 10
Duration of migraine attacks [hours]	21.5 ± 15.5	24.0 (21.5)	2; 48

painkiller intake [days per month]	3.9 ± 3.4	2.5 (1.8)	0; 10
Absenteeism [days per month]	1.2 ± 2.6	0.5 (1.0)	0; 10
Presenteeism [days per month]	3.0 ± 3.2	2.0 (2.0)	0; 10

Table S4. Baseline description of all adherent survey participants with regular migraine.

**Baseline description of adherent survey participants with regular migraine**			
	number	percentage	
Sample size	41	100%	
Female participants	39	95.1%	
Participants with frequent medical care with GP/neurologist	26	63.4%	
Participants with concomitant tension-type headache	25	61.0%	
Participants with prophylactic medication (beta blocker, amitriptylin, flunarizin, topiramat, valproate)	4	9.8%	
Participants with CGRP antibody treatment	1	2.4%	
Participants with Magnesium intake	11	26.8%	
Participants with painkiller intake (NSAR, metamizol, paracetamol)	32	78.0%	
Participants with intake of triptanes	16	39.0%	
	mean ± SD	median (IQR)	min; max
Age [years]	44.3 ± 10.2	47.0 (16.0)	23; 62
Body Mass Index [kg/m2]	27.6 ± 5.2	27.6 (4.5)	18.6; 43.1
Time of diagnosed migraine disease [years]	21.7 ± 11.3	22.0 (20.0)	5; 42
Time between program participation and survey [weeks]	60.6 ± 29.2	63.4 (42.3)	5.7; 114.9
Frequency of migraine [days per month]	5.0 ± 5.3	3.0 (3.3)	2; 28
Average pain level [1-10]	7.4 ± 1.5	7.0 (1.0)	3; 10

Duration of migraine attacks [hours]	29.8 ± 26.8	20.0 (40.0)	1; 96
painkiller intake [days per month]	4.6 ± 4.7	2.0 (5.0)	0; 20
Absenteeism [days per month]	1.3 ± 1.6	0.5 (2.0)	0; 5
Presenteeism [days per month]	4.3 ± 4.8	3.0 (3.0)	0; 28

Table S5. Baseline description of all non-adherent survey participants with rare migraine.

**Baseline description of non-adherent survey participants with rare migraine**			
	number	percentage	
Sample size	12	100%	
Female participants	8	66.7%	
Participants with frequent medical care with GP/neurologist	7	58.3%	
Participants with concomitant tension-type headache	5	41.7%	
Participants with prophylactic medication (beta blocker, amitriptylin, flunarizin, topiramat, valproate)	1	8.3%	
Participants with CGRP antibody treatment	0	0.0%	
Participants with Magnesium intake	0	0.0%	
Participants with painkiller intake (NSAR, metamizol, paracetamol)	11	91.7%	
Participants with intake of triptanes	3	25.0%	
	mean ± SD	median (IQR)	min; max
Age [years]	39.9 ± 10.2	40.0 (14.5)	26; 58
Body Mass Index [kg/m2]	27.3 ± 9.5	24.1 (4.8)	19.3; 50.6
Time of diagnosed migraine disease [years]	14.0 ± 8.6	10.0 (10.3)	5; 31
Time between program participation and survey [weeks]	58.9 ± 25.4	58.6 (33.3)	21.6; 109.3
Frequency of migraine [days per month]	1.0 ± 0.0	1.0 (0.0)	1; 1

Average pain level [1-10]	6.6 ± 1.6	7.0 (2.3)	4; 9
Duration of migraine attacks [hours]	18.8 ± 21.5	6.5 (26.0)	3; 62
painkiller intake [days per month]	1.6 ± 2.8	1.0 (0.5)	0; 10
Absenteeism [days per month]	0.6 ± 1.0	0.0 (1.0)	0; 3
Presenteeism [days per month]	1.8 ± 2.4	1.0 (0.5)	0; 8

Table S6. Baseline description of all adherent survey participants with rare migraine.

**Baseline description of adherent survey participants with rare migraine**			
	number	percentage	
Sample size	17	100%	
Female participants	17	100.0%	
Participants with frequent medical care with GP/neurologist	7	41.2%	
Participants with concomitant tension-type headache	10	58.8%	
Participants with prophylactic medication (beta blocker, amitriptylin, flunarizin, topiramat, valproate)	1	5.9%	
Participants with CGRP antibody treatment	0	0.0%	
Participants with Magnesium intake	1	5.9%	
Participants with painkiller intake (NSAR, metamizol, paracetamol)	16	94.1%	
Participants with intake of triptanes	2	11.8%	
	mean ± SD	median (IQR)	min; max
Age [years]	40.8 ± 11.8	42.0 (18.0)	20; 61
Body Mass Index [kg/m2]	29.4 ± 7.5	29.1 (9.1)	19.7; 43.3
Time of diagnosed migraine disease [years]	18.6 ± 11.8	16.0 (18.0)	1; 40
Time between program participation and survey [weeks]	43.8 ± 23.0	46.7 (27.3)	5.9; 86.9

Frequency of migraine [days per month]	1.0 ± 0.0	1.0 (0.0)	1; 1
Average pain level [1-10]	7.4 ± 1.7	8.0 (2.3)	4; 10
Duration of migraine attacks [hours]	5.5 ± 3.6	5.0 (2.0)	1; 15
painkiller intake [days per month]	1.1 ± 0.8	1.0 (0.0)	0; 4
Absenteeism [days per month]	0.4 ± 0.5	0.0 (1.0)	0; 1
Presenteeism [days per month]	1.3 ± 1.1	1.0 (0.0)	0; 1

Table S7. Description of symptoms after program participation for non-adherent patients with rare migraine

All survey participants without adherence to the nutritional recommendations and rare migraine at baseline (=1 migraine day per month) (n=14) reported migraine symptoms at the time of survey (i.e. after program participations, TP1) and recalled migraine symptoms from the the time before programm participation (TP0). Reported symptoms at TP1 and the change of symptoms from TP0 to TP1 are presented. Data presented as mean, standard deviation (SD), median, interquartile range (IQR), minimum (min), and maximum (max).

Description of symptoms after program participation for non-adherent patients with rare migraine			
Sample size n=12	mean ± SD	median (IQR)	min; max
Frequency of migraine [days per month]	1.9 ± 2.6	1.0 (0.3)	1; 10
change from TP0 to TP1	0.9 ± 2.6	0.0 (0.3)	0; 9
Pain level [1-10]	6.4 ± 1.8	6.5 (1.5)	3; 9
change from TP0 to TP1	- 0.2 ± 0.9	0.0 (0.3)	- 2; 3
Duration of migraine attacks [hours]	18.4 ± 22.5	6.0 (23.3)	3; 72
change from TP0 to TP1	- 0.3 ± 10.7	0.0 (0.0)	- 26; 24
painkiller intake [days per month]	0.6 ± 0.7	1.0 (1.0)	0; 2
change from TP0 to TP1	- 1.0 ± 3.0	0.0 (0.5)	- 10; 1
Absenteeism [days per month]	0.8 ± 1.2	0.0 (1.3)	0; 3
change from TP0 to TP1	0.2 ± 0.9	0.0 (0.0)	- 1; 3

Presenteeism [days per month]	1.9 ± 2.3	1.0 (1.0)	0; 8
change from TP0 to TP1	0.2 ± 0.8	0.0 (0.0)	- 1; 2

Table S8. Description of symptoms after program participation for adherent patients with rare migraine

All survey participants with adherence to the nutritional recommendations and rare migraine at baseline (=1 migraine day per month) (n=17) reported migraine symptoms at the time of survey (i.e. after program participations, TP1) and recalled migraine symptoms from the the time before programm participation (TP0). Reported symptoms at TP1 and the change of symptoms from TP0 to TP1 are presented. Data presented as mean, standard deviation (SD), median, interquartile range (IQR), minimum (min), and maximum (max)

Description of symptoms after program participation for adherent patients with rare migraine			
Sample size n=17	mean ± SD	median (IQR)	min; max
Frequency of migraine [days per month]	1.4 ± 0.9	1.0 (0.0)	0; 4
change from TP0 to TP1	0.4 ± 0.9	0.0 (0.0)	- 1; 3
Pain level [1-10]	6.4 ± 2.4	7.0 (3.0)	1; 10
change from TP0 to TP1	- 1.0 ± 2.2	0.0 (2.0)	- 6; 2
Duration of migraine attacks [hours]	4.9 ± 3.2	4.5 (5.3)	1; 12
change from TP0 to TP1	- 0.8 ± 2.3	0.0 (1.3)	- 7; 4
painkiller intake [days per month]	1.1 ± 0.6	1.0 (0.0)	0; 2
change from TP0 to TP1	- 0.1 ± 1.1	0.0 (0.0)	- 3; 2
Absenteeism [days per month]	0.5 ± 0.7	0.0 (1.0)	0; 2
change from TP0 to TP1	0.1 ± 0.7	0.0 (0.0)	- 1; 2
Presenteeism [days per month]	1.2 ± 1.2	1.0 (0.3)	0; 4
change from TP0 to TP1	0.1 ± 1.5	0.0 (0.3)	- 4; 3
